# Fast walking is a preventive factor against new-onset diabetes mellitus in a large cohort from a Japanese general population

**DOI:** 10.1038/s41598-020-80572-y

**Published:** 2021-01-12

**Authors:** Mariko Iwasaki, Akihiro Kudo, Koichi Asahi, Noritaka Machii, Kunitoshi Iseki, Hiroaki Satoh, Toshiki Moriyama, Kunihiro Yamagata, Kazuhiko Tsuruya, Shouichi Fujimoto, Ichiei Narita, Tsuneo Konta, Masahide Kondo, Yugo Shibagaki, Masato Kasahara, Tsuyoshi Watanabe, Michio Shimabukuro

**Affiliations:** 1 Department of Diabetes, Endocrinology and Metabolism, School of Medicine, Fukushima Medical University, 1 Hikarigaoka, Fukushima City, Fukushima, 960-1295 Japan; 2 Steering Committee of Research On Design of the Comprehensive Health Care System for Chronic Kidney Disease (CKD) Based On the Individual Risk Assessment By Specific, Fukushima, 960-1295 Japan

**Keywords:** Type 2 diabetes, Preventive medicine

## Abstract

Based on questionnaires from 197,825 non-diabetic participants in a large Japanese cohort, we determined impact of (1) habit of exercise, (2) habit of active physical activity (PA) and (3) walking pace on new-onset of type 2 diabetes mellitus. Unadjusted and multivariable-adjusted logistic regression models were used to determine the odds ratio of new-onset diabetes mellitus incidence in a 3-year follow-up. There were two major findings. First, habits of exercise and active PA were positively associated with incidence of diabetes mellitus. Second, fast walking, even after adjusting for multiple covariates, was associated with low incidence of diabetes mellitus. In the subgroup analysis, the association was also observed in participants aged ≥ 65 years, in men, and in those with a body mass index ≥ 25. Results suggest that fast walking is a simple and independent preventive factor for new-onset of diabetes mellitus in the health check-up and guidance system in Japan. Future studies may be warranted to verify whether interventions involving walking pace can reduce the onset of diabetes in a nation-wide scale.

## Introduction

Physical inactivity is closely associated with the incidence of new-onset diabetes^1–3^. In meta-analyses of cohort or randomized studies, an inverse association between physical activity (PA) and risk of type 2 diabetes was observed regardless of intensity (low, moderate or vigorous), total time, and type (leisure-time physical activity (LTPA) or walking) of PA^1^. Smith et al. reported that the inverse relationship between the amount of LTPA (metabolic equivalent task score: METs h/week) and type 2 diabetes was curvilinear^2^. Frequency and intensity of PA and exercise are attributable to physical fitness (PF)^4,5^; therefore, poor physical fitness can also be a risk factor for new-onset diabetes. Walking pace is assumed to be an indicator of PF^6,7^. Mozaffarian et al. reported that a combination score of walking pace and LTPA predicted the 10-year incidence of diabetes mellitus in US participants aged 65 years or older^6^. Joseph et al. reported that diabetes risk was lower in participants with brisk or striding walking pace compared with casual walking pace in a Multi-Ethnic Study of Atherosclerosis participants^7^. Thereby, in official guidelines, promotion of mild and moderate to vigorous PA has been recommended as a prevention and treatment option for diabetes mellitus^8–10^.

In Japan, a nationwide health check-up and guidance system called “the Specific Health Check and Guidance System (SHCG)” has been operated since 2008^11^. The SHCG has been focused on prevention of metabolic syndrome (MetS) and its related disorders such as type 2 diabetes mellitus and health guidance including the promotion of PA/exercise has been provided for individuals who have abdominal obesity and/or additional MetS risk factors. Theoretically, such health guidance can be effective in the nationwide participants with a high MetS risk for preventing new-onset of type 2 diabetes mellitus. In the SHCG, all participants were administered three simple questions regarding PA/exercise: (1) habit of regular exercise (≥ 2 times/week of exercise ≥ 4 METs h), (2) habit of active PA (≥ 23 **ETs h/week) and (3) walking pace (rapid or not rapid), an indicator of physical fitness. We hypothesized that responses to the questions can be associated with onset of type 2 diabetes mellitus and if so, what characteristics such as sex, age, and leanness/obesity are linked to the association.

We evaluated the associations of the aforementioned three measures of PA and PF with a 3-year incidence of new-onset diabetes mellitus in a nation-wide Japanese general population. Considering the differences in diabetes incidence and mechanisms associating diabetes with PA, we also evaluated whether the association of PA with incident diabetes mellitus varied by sex, age, and leanness/obesity.

## Results

### General characteristics

Among all participants who did not have diabetes mellitus (n = 577,984) in 2008 (Dataset 1, Additional file 1, The flow chart of the participants' recruitment. those who visited only in 2008 (n = 162,740) or who had missing data (n = 247,560) were excluded. The main analysis was performed on the complete cases (Dataset 3, n = 167,684, Table 1). The mean age was 63.7 years and 38.8% were men. There were 6,229/167,684 (3.7%) patients who had not been diabetic in 2008 and developed diabetes between 2009 and 2011. Frequencies of regular exercise (exercise to sweat lightly) (45.4% vs 41.6%, P < 0.01) and active PA (walking > 1 h/day) (54.1% vs 52.1%, P < 0.01) were significantly higher in patients who developed diabetes compared to non-diabetic patients; however, that of fast walking was lower in the participants who developed diabetes (47.9 vs 50.2%, P < 0.01) (Table 1). Frequency of weight change ± 3 kg within 1 year was higher in the Diabetes onset + group.

**Table 1:** Baseline characteristics of participants.

	Total	Diabetes onset ( −)	Diabetes onset ( +)	*P*
n	167,684	161,455	6,229	
Age, years	63.7 (7.8)	63.6 (7.8)	65.4 (6.5)	< 0.01
% Male	38.8	38.3	51.7	< 0.01
BMI, kg/m^2^	23.0 (3.1)	22.9 (3.1)	24.3 (3.5)	< 0.01
Waist circumference, cm	83.3 (8.8)	83.1 (8.8)	86.8 (9.1)	< 0.01
Systolic blood pressure, mmHg	129 (17)	129 (17)	134 (17)	< 0.01
Diastolic blood pressure, mmHg	76 (11)	76 (11)	78 (11)	< 0.01
Fasting plasma glucose, mg/dl	93.3 (9.7)	92.9 (9.3)	104.8 (11.6)	< 0.01
HbA1c, %	5.59 (0.33)	5.57 (0.32)	6.01 (0.34)	< 0.01
LDL cholesterol, mg/dL	126.6 (29.7)	126.6 (29.7)	126.5 (31.5)	N.S
HDL cholesterol, mg/dL	62.7 (16.0)	62.9 (16.0)	58.3 (15.2)	< 0.01
Triglycerides, mg/dL	112.6 (69.5)	111.8 (68.8)	133.5 (84.5)	< 0.01
AST, U/L	24.0 (9.2)	23.9 (9.0)	25.8 (12.4)	< 0.01
ALT, U/L	21.2 (12.4)	21.1 (12.2)	25.4 (16.4)	< 0.01
ɤGT, U/L	34.2 (40.5)	33.8 (39.9)	44.4 (53.8)	< 0.01
Hypertension, %	44.0	43.4	61.4	< 0.01
Dyslipidemia, %	54.5	54.1	64.9	< 0.01
Current smoker, %	13.1	12.9	16.9	< 0.01
Everyday drinking, %	22.1	22.2	25.6	< 0.01
Weight gain over 10 kg since 20 years of age, %	31.2	30.6	46.2	< 0.01
Weight change ± 3 kg within 1 year, %	19.9	19.7	26.2	< 0.01
Exercise to sweat lightly, %	41.7	41.6	45.4	< 0.01
Walking > 1 h/day, %	52.2	52.1	54.1	< 0.01
Fast walking, %	50.1	50.2	47.9	< 0.01

*AST* aspartate aminotransferase, *ALT* alanine aminotransferase, *γGT* γ-glutamyl transpeptidase, *N.S.* not significant.

Values are Mean (SD) or %. *P*: provability by two-tailed unpaired t-test or χ^2^ test. BMI: body mass index.

### Odds ratio (OR) of new-onset diabetes

**Figure 1:**
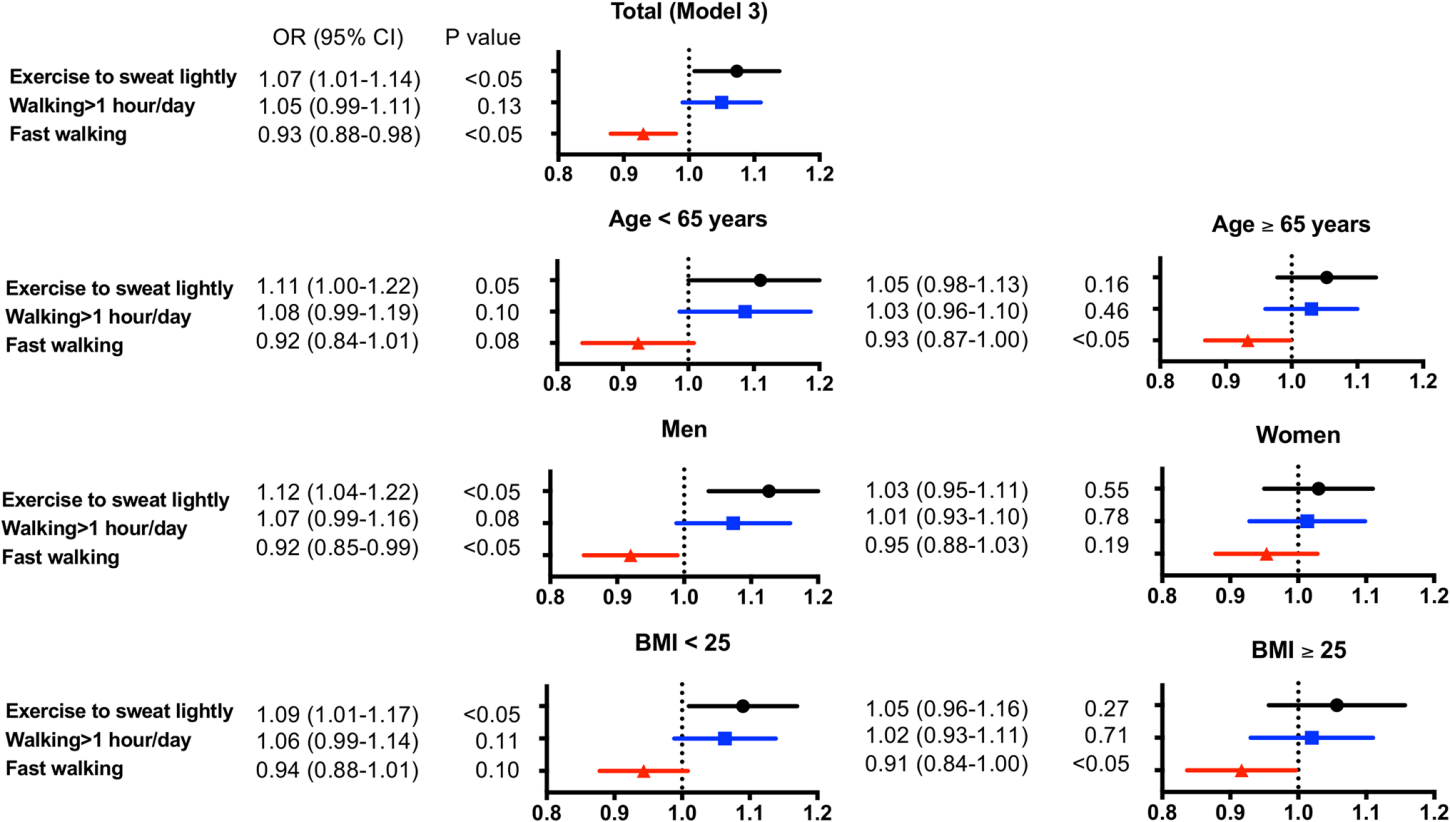
Adjusted odds ratio (OR) for new-onset diabetes mellitus (model 3). Among non-diabetic participants aged 40–74 years (n = 167,684), adjusted ORs (95% confidential intervals) for new-onset diabetes mellitus in the 3-year follow-up were calculated for exercise to sweat lightly (●), walking > 1 h/day (■), and fast walking (▲). Model 3: adjusted for sex, age, body mass index (BMI), systolic blood pressure (SBP), current smoking, drinking, weight gain over 10 kg from 20 years of age, weight change of 3 kg within 1 year, exercise to sweat lightly, walking > 1 h/day and fast walking.

OR for new-onset diabetes due to differences in exercise, PA and walking pace was examined by logistic regression analysis (Table 2). Fast walking was inversely associated with onset of diabetes mellitus (OR 0.93, 95% confidence interval [CI] 0.88–0.98, P < 0.05); however, regular exercise (OR 1.19, 95% CI 1.13–1.26, P < 0.05) and active PA (OR 1.11, 95% CI 1.05–1.17, P < 0.05) (Model 1) were positively associated. After adjusting for sex, age and BMI (Model 2), the association between fast walking and onset of diabetes mellitus did not reach to significance difference. However, after adjusted with multiple factors including two other PA measures "exercise to sweat lightly” and "walking > 1 h/day", fast walking was negatively associated with onset of diabetes (Model 3). Multivariate analysis was performed also in the subgroups: age < 65 vs ≥ 65 years, men vs women, and BMI < 25 vs ≥ 25. Fast walking was negatively associated with onset of diabetes mellitus in patients aged ≥ 65 years, male sex, and patients with BMI ≥ 25 (Fig. 1 and Table 2). Exercise to sweat lightly was positively associated with onset of diabetes after multivariate adjustment (Model 2 and 3) in all and in patients aged < 65, male sex and patients with BMI < 25.

**Table 2:** Unadjusted and multivariable-adjusted odds ratio for the risk of new-onset diabetes mellitus of three physical activity measures.

	Model 1 (Unadjusted)		Model 2		Model 3	
	OR (95% CI)	*P*	OR (95% CI)	*P*	OR (95% CI)	*P*
**All**						
Exercise to sweat lightly	1.19 (1.13–1.26)	< 0.05	1.06 (1.00–1.12)	< 0.05	1.07 (1.01–1.14)	< 0.05
Walking > 1 h/day	1.11 (1.05–1.17)	< 0.05	1.05 (1.00–1.11)	0.06	1.05 (0.99–1.11)	0.13
Fast walking	0.93 (0.88–0.98)	< 0.05	0.96 (0.91–1.02)	0.16	0.93 (0.88–0.98)	< 0.05
**Age < 65 years**						
Exercise to sweat lightly	1.20 (1.09–1.31)	< 0.05	1.10 (1.01–1.21)	< 0.05	1.11 (1.00–1.22)	0.05
Walking > 1 h/day	1.09 (1.00–1.19)	0.06	1.10 (1.01–1.20)	< 0.05	1.08 (0.99–1.19)	0.10
Fast walking	0.91 (0.83–0.99)	< 0.05	0.96 (0.88–1.04)	0.32	0.92 (0.84–1.01)	0.08
**Age ≥ 65 years**						
Exercise to sweat lightly	1.05 (0.99–1.12)	0.11	1.02 (0.96–1.09)	0.48	1.05 (0.98–1.13)	0.16
Walking > 1 h/day	1.02 (0.95–1.08)	< 0.05	1.02 (0.95–1.08)	0.62	1.03 (0.96–1.10)	0.46
Fast walking	0.89 (0.83–0.95)	< 0.05	0.94 (0.88–1.00)	< 0.05	0.93 (0.87–1.00)	< 0.05
**Men**						
Exercise to sweat lightly	1.19 (1.11–1.27)	< 0.05	1.11 (1.03–1.19)	< 0.05	1.12 (1.04–1.22)	< 0.05
Walking > 1 h/day	1.10 (1.03–1.19)	< 0.05	1.08 (1.01–1.16)	< 0.05	1.07 (0.99–1.16)	0.08
Fast walking	0.92 (0.86–0.99)	< 0.05	0.95 (0.88–1.02)	0.14	0.92 (0.85–0.99)	< 0.05
**Women**						
Exercise to sweat lightly	1.06 (0.98–1.14)	0.13	1.00 (0.93–1.08)	0.940	1.03 (0.95–1.11)	0.55
Walking > 1 h/day	1.02 (0.95–1.10)	0.6	1.01 (0.94–1.09)	0.80	1.01 (0.93–1.10)	0.78
Fast walking	0.87 (0.81–0.93)	< 0.05	0.95 (0.88–1.02)	0.18	0.95 (0.88–1.03)	0.19
**BMI < 25**						
Exercise to sweat lightly	1.26 (1.18–1.35)	< 0.05	1.07 (1.01–1.15)	< 0.05	1.09 (1.01–1.17)	< 0.05
Walking > 1 h/day	1.17 (1.09–1.25)	< 0.05	1.06 (1.00–1.14)	0.07	1.06 (0.99–1.14)	0.11
Fast walking	1.01 (0.94–1.07)	0.87	0.96 (0.90–1.03)	0.26	0.94 (0.88–1.01)	0.1
**BMI ≥ 25**						
Exercise to sweat lightly	1.06 (0.97–1.15)	0.18	1.03 (0.94–1.12)	0.54	1.05 (0.96–1.16)	0.27
Walking > 1 h/day	1.02 (0.94–1.11)	< 0.05	1.01 (0.93–1.10)	0.74	1.02 (0.93–1.11)	0.71
Fast walking	0.90 (0.83–0.98)	< 0.05	0.93 (0.85–1.01)	0.08	0.91 (0.84–1.00)	< 0.05

*OR* odds ratio, *CI* confidential interval, *BMI* body mass index, SBP: systolic blood pressure.

Model 2 (sex, age and BMI, if not applicable to sub-group variables). Model 3 (sex, age, BMI, SBP, current smoking, drink, weight gain over 10 kg from 20-years, weight change of 3 kg within 1 year, exercise to sweat lightly, walking > 1 h/day and fast walking, if not applicable to sub-group variables).

### Characteristics of participants with or without fast walking (Table 3)

**Table 3:** Baseline characteristics of participants with or without fast walking.

	Fast walking ( −)	Fast walking ( +)	*P*
n	83,705	83,979	
Age, years	63.3 (8.0)	64.1 (7.5)	< 0.01
Male, %	37.2	40.3	< 0.01
BMI, kg/m^2^	23.2(3.3)	22.7(2.9)	< 0.01
Waist circumference, cm	83.9(9.2)	82.6(8.4)	< 0.01
Systolic blood pressure, mmHg	128.9(17.6)	129.0 (17.3)	N.S
Diastolic blood pressure, mmHg	76.3(10.7)	76.4(10.6)	N.S
Fasting plasma glucose, mg/dl	93.3(9.7)	93.3(9.7)	N.S
HbA1c, %	5.59 (0.33)	5.58 (0.33)	< 0.01
LDL cholesterol, mg/dL	126.5(29.8)	126.7(29.7)	N.S
HDL cholesterol, mg/dL	62.0(15.8)	63.4(16.1)	< 0.01
Triglycerides, mg/dL	112.6(68.2)	109.8(68.3)	< 0.01
AST, U/L	24.0(9.9)	23.9(8.5)	< 0.05
ALT, U/L	21.5(13.2)	20.9(11.6)	< 0.01
ɤGT, U/L	34.4(41.4)	34.1(39.6)	N.S
Hypertension, %	45.0	43.1	< 0.01
Dyslipidemia, %	54.5	54.4	N.S
Current smoker, %	13.8	12.3	< 0.01
Everyday drinking, %	20.4	23.7	< 0.01
Weight gain over 10 kg since 20 years of age, %	32.9	29.6	< 0.01
Weight change ± 3 kg within 1 year, %	20.3	19.5	< 0.01

Values are Mean (SD) or %. *P* provability by two-tailed unpaired *t* test or χ^2^ test. *BMI* body mass index, *AST* aspartate aminotransferase, *ALT* alanine aminotransferase, *γGT* γ-glutamyl transpeptidase, *N.S.* not significant.

At baseline, BMI and waist circumference were lower, whereas age and the proportion of male participants were higher in fast walking + group compared to those in fast walking—(Table 3). HbA1c was slightly lower, and other metabolic parameters in the fast walking + group were: HDL level was higher, and levels of triglycerides, aspartate aminotransferase, and alanine aminotransferase were lower than in the fast walking-group. The frequency of hypertension and current smokers were lower, and frequency of regular drinkers was higher in the fast walking + group. Frequency of weight change ± 3 kg within 1 year was slightly lower in the fast walking + group.

In a subgroup of our participants (n = 183), we investigated whether self-reported walking speed using the questionnaire was related to objectively measured walking speed (10 meters walking speed, sec). Fast walking + group (n = 69) showed a higher speed than fast walking – group (n = 114) [6.20 ± 1.11 sec/10 m (6.0 ± 1.10 kph) vs 7.08 ± 2.11 sec/10 m (5.4 ± 1.19 kph), P = 0.002 (Additional File 2)].

### Characteristics of participants with or without exercise to sweat lightly (Additional File 3) and with or without walking > 1 h/day (Additional File 4)

In participants with habits of exercise to sweat lightly (Additional File 3) or with walking > 1 h/day (Additional File 4), male gender was higher and fasting plasma glucose was slightly higher.

### Changes in characteristics in three physical activity measures subgroups (Table 4)

**Table 4:** Characteristics of three physical activity measures subgroups at baseline and follow-up or onset of diabetes.

	Fast walking (−)						Fast walking (+)					
	Diabetes onset (−)			Diabetes onset (+)			Diabetes onset (−)			Diabetes onset (+)		
	Baseline	Follow-up	*P*	Baseline	At onset	*P*	Baseline	Follow-up	*P*	Baseline	At onset	*P*
n	80,457			3,247			80,995			2,982		
BMI, kg/m^2^	23.2 (3.3)	23.2 (3.3)	0.488	24.7 (3.7)**	25.0 (3.9)	<0.05	22.69 (2.89)	22.70 (2.92)	<0.05	23.9 (3.1)**	24.2 (3.3)	<0.05
Waist circumference, cm	83.8 (9.1)	83.8 (9.2)	0.35	87.8 (9.5)**	88.4 (9.9)	<0.05	82.5 (8.4)	82.5 (8.4)	0.95	85.6 (8.5)**	86.3 (8.8)	<0.05
Fasting plasma glucose, mg/dl	92.9 (9.3)	92.9 (9.8)	0.53	104.4 (11.7)**	117.3 (24.0)	<0.05	93.0 (9.3)	93.1 (9.8)	<0.05	105.9 (11.4)**	118.7 (22.7)	<0.05
HbA1c, %	5.57 (0.32)	5.59 (0.32)	<0.05	6.00 (0.34)**	6.35 (0.66)	<0.05	5.57 (0.32)	5.59 (0.32)	<0.05	6.02 (0.33)**	6.36 (0.62)	<0.05
Body weight, kg	56.9 (10.2)	56.8 (10.2)	<0.05	61.4 (11.4)**	62.0 (11.8)	<0.05	56.6 (9.8)	56.5 (9.9)	<0.05	60.4 (10.2)**	61.0 (10.6)	<0.05
Exercise to sweat lightly, %	29.6	–	–	33.9**	–	–	53.4	–	–	57.5**	–	–
Walking>1 hour/day, %	40.9	–	–	43.7**	–	–	63.3	–	–	65.6*	–	–
Fast walking, %	0	–	–	0	–	–	100	–	–	100	–	–
Weight change ± 3kg within 1year, %	20.0	–	–	26.8**	–	–	19.3	–	–	25.6**	–	–

	Exercise to sweat lightly (−)						Exercise to sweat lightly (+)					
	Diabetes onset (−)			Diabetes onset (+)			Diabetes onset (−)			Diabetes onset (+)		
	Baseline	Follow-up	*P*	Baseline	At onset	*P*	Baseline	Follow-up	*P*	Baseline	At onset	*P*
n	94,366			3,404			67,086			2,825		
BMI, kg/m^2^	22.9 (3.2)	22.9 (3.2)	0.348	24.6 (3.7)**	24.8 (3.8)	<0.05	23.0 (2.9)	23.0 (3.0)	0.056	24.1 (3.2)**	24.3 (3.4)	<0.05
Waist circumference, cm	83.14 (9.09)	83.11 (9.09)	0.06	87.4 (9.5)**	88.1 (9.9)	<0.05	83.1 (8.3)	83.1 (8.4)	0.23	86.0 (8.5)**	86.6 (8.8)	<0.05
Fasting plasma glucose, mg/dl	92.7 (9.3)	92.7 (9.7)	0.4	104.7 (11.5)**	118.1 (24.2)	<0.05	93.3 (9.3)	93.4 (9.9)	<0.05	105.5 (11.6)**	117.8 (22.4)	<0.05
HbA1c, %	5.56 (0.32)	5.58 (0.32)	<0.05	6.01 (0.34)**	6.36 (0.66)	<0.05	5.58 (0.31)	5.60 (0.32)	<0.05	6.01 (0.34)**	6.34 (0.61)	<0.05
Body weight, kg	56.6 (10.2)	56.5 (10.3)	<0.05	61.1 (11.3)**	61.7 (11.7)	<0.05	57.0 (9.7)	56.8 (9.7)	<0.05	60.7 (10.3)**	61.2 (10.7)	<0.05
Exercise to sweat lightly, %	0	–	–	0	–	–	100	–	–	100	–	–
Walking>1 hour/day, %	36.2	–	–	35.5**	–	–	74.4	–	–	76.6*	–	–
Fast walking, %	40.0	–	–	37.0**	–	–	64.5	–	–	61.0**	–	–
Weight change ± 3kg within 1year, %	20.3	–	–	26.9**	–	–	18.8	–	–	25.5**	–	–

	Walking>1 hour/day (−)						Walking>1 hour/day (+)					
	Diabetes onset (−)			Diabetes onset (+)			Diabetes onset (−)			Diabetes onset (+)		
	Baseline	Follow-up	*P*	Baseline	At onset	*P*	Baseline	Follow-up	*P*	Baseline	At onset	*P*
n	77,321			2,856			84,131			3,373		
BMI, kg/m^2^	23.0 (3.2)	23.0 (3.2)	0.9	24.6 (3.6)**	24.9 (3.8)	<0.05	22.9 (3.0)	22.9 (3.0)	<0.05	24.1 (3.3)**	24.4 (3.5)	<0.05
Waist circumference, cm	83.44 (9.05)	83.39 (9.09)	<0.05	87.6 (9.3)**	88.3 (9.7)	<0.05	82.8 (8.5)	82.9 (8.6)	0.09	86.1 (8.9)**	86.7 (9.2)	<0.05
Fasting plasma glucose, mg/dl	92.7 (9.3)	92.8 (9.7)	<0.05	104.5 (11.7)**	118.2 (24.7)	<0.05	93.1 (9.3)	93.2 (9.8)	<0.05	105.6 (11.5)**	117.8 (22.3)	<0.05
HbA1c, %	5.57 (0.32)	5.58 (0.32)	<0.05	6.01 (0.34)**	6.35 (0.61)	<0.05	5.57 (0.31)	5.59 (0.32)	<0.05	6.01 (0.34)**	6.35 (0.66)	<0.05
Body weight, kg	57.0 (10.2)	56.8 (10.3)	<0.05	61.3 (11.2)**	62.0 (11.6)	<0.05	56.6 (9.7)	56.5 (9.9)	<0.05	60.6 (10.5)**	61.1 (10.9)	<0.05
Exercise to sweat lightly, %	22.2	–	–	23.1**	–	–	59.4	–	–	64.2**	–	–
Walking>1 hour/day, %	0	–	–	0	–	–	100	–	–	100	–	–
Fast walking, %	38.5	–	–	36.0**	–	–	60.9	–	–	58.0**	–	–
Weight change ± 3kg within 1year, %	20.4	–	–	27.6**	–	–	19.0	–	–	25.1**	–	–

In subgroups with or without three physical activity measures, BMI, waist circumference, fasting plasma glucose, HbA1c and body weight and frequency of weight change ± 3 kg within 1 year were higher in Diabetes onset + than in Diabetes onset-group (Table 4). In all six subgroups, BMI and waist circumference did not change or changed very slightly at follow-up in Diabetes onset-group, but significantly increased at onset of diabetes in Diabetes onset + group. In all participants, BMI and waist circumference were increased in Diabetes onset + , but not in Diabetes onset – group (Additional File 5).

## Discussion

This study revealed two major findings regarding the association between PA measures (habit of exercise, habit of active PA and walking pace) and the incidence of diabetes mellitus in a large Japanese cohort. First, habit of exercise or active PA was positively associated with incidence of diabetes mellitus. Second, fast walking, even after adjustment with multiple covariates, was associated with low incidence of diabetes mellitus and the association was also observed in participants aged ≥ 65 years, in men, and in those with a body mass index ≥ 25 (Table 2, Model 3).

By using the questionnaire integrating PA (habit of exercise and habit of active PA) and physical fitness (walking pace), this study evaluated the association between PA components and the incidence of diabetes mellitus. Helmrich et al. reported that weekly amounts of PA (physical activity index, kcal/week) were associated with a reduced risk for type 2 diabetes in the study assessing PA by questionnaires in 5,990 male alumni of the University of Pennsylvania^12^. Hu et al. examined the risk of developing type 2 diabetes in 70,102 female nurses aged 40–65 years and reported that the risk of type 2 diabetes was reduced stepwise by the magnitude of weekly PA (MET) regardless of the type of PA (walking, jogging, running, bicycling, calisthenics, aerobics, aerobic dance, rowing machine, lap swimming, squash or racquetball and tennis)^13^.

In this study, habit of regular exercise (defined as ≥ 2 times/week of exercise ≥ 4 METs•h, which indicates moderate to vigorous PA (MVPA)), was not associated with prevention of diabetes mellitus. At least two explanations can be raised for the discrepancy between our study and previous studies^12,13^ on the association of MVPA and onset of diabetes. First, the question in our study was too simple and we could not determine the weekly amounts of PA; thus, limiting the power of the study for estimating onset of diabetes mellitus. In the Nurses’ Health Study, multivariate relative ratios (RRs) of type 2 diabetes were decreased stepwise from 1.0 to 0.58 across quintiles of total PA/week (P for trend < 0.001)^13^, indicating that amount of PA was associated with the risk of diabetes onset. In this study, regular active PA, equivalent to walking twice per week, did not have the power to estimate onset of diabetes. Collectively, frequency and intensity, not presence or absence, of regular active PA may be required to estimate new-onset of diabetes. Second, the participants with habits of exercise and/or active PA in our study could be a high-risk population (Additional Files 3 and 4). In the SHCG, a recommendation has been provided on exercise and PA to prevent or reduce the incidence of metabolic syndrome. Thus, our participants with waist circumference ≥ 85 cm in men or ≥ 90 cm in women^14,15^ or with fasting plasma glucose ≥ 100 mg/dL could have been encouraged more for regular exercise or active PA. Actually, the participants with habits of regular exercise and/or active PA showed slightly higher values of fasting plasma glucose (Additional Files 3 and 4).

The questions on habits of regular walking had no power to determine low incidence of diabetes mellitus; however, that on fast walking pace had a strong power to do so. There are two possible explanations. First, walking speed may be critical for preventing onset of diabetes. In the Nurses’ Health Study, multivariate RRs were 0.86 (95% CI 0.73–1.01) for “normal or average (3.2–4.8 km/h)” usual walking pace and 0.59 (95% CI 0.47–0.73) for “brisk (4.8–6.2 km/h) or striding (6.4 km/h or faster)” pace compared to women with “easy or casual (less than 3.2 km/h)” pace^13^. Fast walking is estimated to be 3.8 METs and thus categorized as exercise of moderate intensity (3.0–6.0 MET)^16^. It has been reported that RRs for exercise of moderate intensity was 0.83 (95% CI 0.75–0.91)^17,18^, equivalent to that of normal to brisk walking^16^. Combined, walking speed, not presence or absence of regular walking, is efficient to estimate new-onset of diabetes. Second, fast walking can be an indicator of the low-risk group for onset of diabetes discussed below.

There are three explanations why fast walking is an indicator of the low-risk group for onset of diabetes. First, habit of intentional fast walking may reduce onset of diabetes. Theoretically, METs are higher in fast walking, as compared to non-fasting walking; thus, maintenance of fast walking can be protective for the onset of diabetes by increasing daily METs^17^. Following the guidance in the Specific Health Check and Guidance System, regular exercise and/or active PA had been recommended to our participants who were at or had a risk(s) for metabolic syndrome^14^. However, in the guidance for walking, frequency (3 days/week) and duration (20 min plus per day), but not pace of walking, have been recommended; thus, it is not likely that a habit of intentional fast walking was linked to onset of diabetes in our participants.

Second, fast walking may reflect a high level of physical fitness, which could be protective against new-onset of diabetes. Self-reported walking speed was closely related to objectively measured walking speed among community-dwelling older people^19^. In our subgroup, self-reported walking speed using the questionnaire was related to the objectively measured walking speed (10 m walking speed, sec)^20,21^. Fast walking + vs − showed larger differences in BMI and waist circumference (Table 3) as compared to exercise to sweat lightly + vs − or walking > 1 h/day + vs − . A higher METs in subjects with fast walking + might be protective^22,23^ against the onset of obesity and/or diabetes as compared to regular exercise or physical activity. BMI and waist circumference were increased only in Diabetes onset + , but not in Diabetes onset – in all three measures subgroups (Table 4). In Model 3, fast walking was not significanty associated with onset of diabetes when deleted other two physical activities (data not shown). These three physical activity measures can be linked to onset of diabetes in a mutually dependent manner.

Third, fast walking may represent a factor in inhibiting onset of diabetes besides physical fitness. In our study, BMI and waist circumference were lower, and age, male sex and frequency of regular drinking were higher in the fast walking + group (Table 3). According to a survey on leisure activities targeting adults over the age of 18 years in Michigan, USA, significant characteristics of fast walkers (≥ 5.6 kph) were men, had high educational background and high annual income, and were also associated with being a smoker and had a high frequency of alcohol consumption^24^, exhibiting very similar characteristics to our fast walking + group. Although smoking and alcohol drinking are not factors protecting against diabetes, a low BMI can work as a protective factor. Reportedly, low BMI is a strong predictor of habitual exercise^25,26^; thus, low adiposity can protect ones from diabetes through exercise-induced increase in muscle insulin sensitivity^27^. Actually, walking pace showed correlations with participation in higher intensity PA, high volumes of total non-occupational PA, and higher frequency and total walking volume^24,28^. However, low BMI is thought to be a low risk factor for diabetes due to high insulin sensitivity regardless of exercise habits^29^. In fast walking + group, frequencies of weight gain over 10 kg from twenty and weight change ± 3 kg within 1 year were also lower, suggesting that at least partly fast walking is an indicator for low fluctuation of body weight. Meanwhile, in both subgroups with or without weight gain over 10 kg from twenty, and in without weight change ± 3 kg within 1-year, fast walking was negatively associated with onset of diabetes (Additional File 6). Considering all of the above, fast walking may be a suppressor of diabetes onset regardless of whether or not there is a history of weight gain.

There are a number of studies that have investigated the prevention of the onset of type 2 diabetes by intervention in lifestyle habits including exercise therapy. The relative risk reduction (RRR) of diabetes onset in the study intervention groups such as the Finish Diabetes Prevention Study^30,31^, Diabetes Prevention Program Research Group^32^, Kosaka et al.^33^, China Da Qing Diabetes Prevention Study^34^, and Indian Diabetes Prevention Programme^35^ were 58%, 58%, 67%, 51% and 29%, respectively, compared with the control groups. Results of meta-analysis have also shown a preventive effect on nearly half of the subjects, with a RRR of 49%^36^. However, appropriate assessment scale of current habits for PA/exercise and appropriate personalized goals has not been clarified for preventing type 2 diabetes mellitus in a nation-wide scale. This study suggests that fast walking is a simple and independent preventive factor for new-onset of diabetes mellitus. It may be required to verify that the intervention of walking pace is effective to reduce onset of diabetes in future studies.

This study has several limitations. First, in the specific health examination of Japanese citizens aged 40–74 years, there were as many as 51.91 million people from March 2008 to April 2009. However, examination is not an obligation; thus, only 20.01 million people (37.4%) were examined. Therefore, this study may have a bias. Second, because of the age range of 40–74 years old, the onset of diabetes before 39 years old is unknown. Therefore, it will not be a factor in the analysis of juvenile onset type 2 diabetes. Third, the information recorded on the questionnaires was self-reported and judgment on walking pace was subjective. Fourth, the observation period was short. Fifth, we could determine only “self-reported”, but not real, timing of last meal, suggesting that little non-fasting (< 10 h) glucose may be included in the analysis. Sixth, because comparing group difference for large samples could link to type I error, we should be careful to interpret true differences between groups.

In conclusion, fast walking is a simple and independent preventive factor for new-onset of diabetes mellitus in the health check-up and guidance system in Japan. It is necessary to verify whether intervention of walking pace reduces onset of diabetes in future studies.

## Methods

### Study population

This study was a cohort study using data of the annual health check program, “The Specific Health Check and Guidance System” (SHCG) in Japan^37–39^, launched by the Ministry of Health, Labor and Welfare, Japan in 2008. The target of SHCG was the Japanese general population between the ages of 40 and 74 years, estimated to be 51,919,920 at the beginning of 2008. This study was performed as a part of the ongoing project “Design of the comprehensive healthcare system for chronic kidney disease (CKD) based on the individual risk assessment by specific health checkups.” The completed STROBE checklist was provided as Additional file 7.

Twenty-seven of Japan’s 47 prefectural governments (Hokkaido, Miyagi, Yamagata, Fukushima, Ibaraki, Tochigi, Tokyo, Saitama, Chiba, Kanagawa, Niigata, Nagano, Ishikawa, Fukui, Gifu, Osaka, Hyogo, Okayama, Tokushima, Kochi, Fukuoka, Saga, Nagasaki, Oita, Kumamoto, Miyazaki, and Okinawa) had agreed to participate in the study and the residents were included in the analysis. The individual data of the SHCG from 2008 to 2011 had been sent to and verified by an independent data center, the Japan Clinical Research Support Unit (Tokyo, Japan), which is a non-profit organization^38^. The community approval was obtained from prefecture representatives.

Among the participants from the 27 prefectures, we excluded those who visited only once in 2008 and those with incomplete information recorded in the database, such as data on sex, age, body mass index (BMI), systolic (SBP) and diastolic blood pressure (DBP), fasting plasma glucose (FPG) levels, HbA1c, and regular exercise. We finally selected 167,684 without diabetes mellitus from 691,475 participants (see definition below) at baseline 2008.

### Measurements

Trained staff measured height, body weight, blood pressure, and waist circumference of each subject. Questionnaires were administered to record data on smoking status (current smoker or not), drinking habits (everyday, sometimes, rarely or never), regular exercise (exercise to sweat lightly for over 30 min on each occasion, two times weekly, walking > 1 h/day, fast walking), anti-hypertensive drug use, anti-hyperglycemic drug use, and lipid-lowering drug use. Fasting blood samples were collected after an overnight fast for ≥ 10 h (In Japanese; https://www.mhlw.go.jp/file/05-Shingikai-12401000-Hokenkyoku-Soumuka/0000158929.pdf) and were assayed within 24 h with automatic clinical chemical analyzers. We excluded participants who were not available for fasting blood samples. When needed, HbA1c was corrected as a National Glycohemoglobin Standardization Program equivalent value and calculated using the following formula: HbA1c (%) = HbA1c (Japan Diabetes Society) (%) + 0.4%^40^.

In a subgroup of participants (n = 183), we evaluated the association between self-reported walking speed on the questionnaire and objectively measured walking speed. The time required for walking 10 meters was measured by modifications^20,21^ and the body composition was assessed by a body composition analyzer (InBody 770, InBody, Seoul, Korea)^41^ based on the segmental multi-frequency bioelectrical impedance analysis (SMF-BIA)^42^.

### Definition of diabetes mellitus, dyslipidemia and hypertension

A participant was recognized as diabetes mellitus, when fasting plasma glucose level was ≥ 126 mg/dL, or the HbA1c level was ≥ 6.5% (48 mmol/mol), or the participant had a regular use of anti-hyperglycemic drugs at baseline (2008). Participants without diabetes mellitus at 2008 were followed up for fasting plasma glucose and HbA1c at 2009, 2010 and 2011. If ones met any one of the above diabetes criteria, we defined them as new-onset diabetes mellitus. A participant was recognized hypertension, if SBP was ≥ 140 mmHg, or if DBP was ≥ 90 mmHg, or if she/he had a regular use of antihypertensive drugs. A participant was recognized dyslipidemia if high-density lipoprotein (HDL)-C levels were < 40 mg/dL (1.0 mmol/L), if low-density lipoprotein-cholesterol levels were ≥ 140 mg/dL (3.6 mmol/L), or if triglyceride levels were ≥ 150 mg/dL (1.7 mmol/L), or if they had a regular use of lipid-lowering drugs.

### Statistical analyses

Two-tailed paired or unpaired t-test was used for group means comparison. χ2 test or McNemar test were used for unpaired or paired comparisons of two categorical variables. Unadjusted and multi-variate adjusted logistic regression models were used to evaluate association between three physical activity measures (exercise to sweat lightly, walking > 1 h/day and fast walking) at baseline and new-onset diabetes mellitus. First, we performed unadjusted analyses (Table 2, Model 1), adjusted for sex, age, and BMI (Model 2). Finally, we adjusted the model for sex, age, BMI, SBP, current smoking, drink, weight gain over 10 kg from 20-years, weight change of 3 kg within 1 year, exercise to sweat lightly, walking > 1 h/day and fast walking (Model 3). All analyses were performed by SPSS software (version 24.0; SPSS, Chicago, IL, USA).

### Ethics approval and consent to participate

The research protocol had been approved by the Ethics Committee of Fukushima Medical University (#1485 and #2771) and all procedures performed in the studies involving human participants were conducted in accordance with its ethical standards and with the 1964 Helsinki declaration and its later amendments or com-parable ethical standards. This study was performed also according to the Ethical Guidelines for Medical and Health Research Involving Human Subjects enacted by the Ministry of Health, Labour and Welfare of Japan (http://www.mhlw.go.jp/file/06-Seisakujouhou-10600000-Daijinkanboukouseikagakuka/0000069410.pdf; http://www.mhlw.go.jp/file/06-Seisakujouhou-10600000-Daijinkanboukouseikagakuka/0000080278.pdf). Informed Consent was waived by the Ethics Committee of Fukushima Medical University. Instead, we publicized information concerning this study on the web (http://www.fmu.ac.jp/univ/sangaku/data/koukai_2/2771.pdf) and ensured that the subjects could refuse the use of their personal information.

The study protocol to measure objectively measured walking speed was approved by the Fukushima Medical University Ethics Committee (Number 29118). The written informed consent was taken from all patients in the subgroup analysis.

## Supplementary Information

Supplementary Information.

Supplementary Information.

Supplementary Information.

Supplementary Information.

Supplementary Information.

Supplementary Information.

Supplementary Information.

## Data Availability

The datasets used and analyzed during the current study are available from the corresponding author on reasonable request.

## References

[1] Aune D, Norat T, Leitzmann M, Tonstad S, Vatten LJ (2015). Physical activity and the risk of type 2 diabetes: a systematic review and dose-response meta-analysis. Eur. J. Epidemiol..

[2] Smith AD, Crippa A, Woodcock J, Brage S (2016). Physical activity and incident type 2 diabetes mellitus: a systematic review and dose-response meta-analysis of prospective cohort studies. Diabetologia.

[3] Patterson R (2018). Sedentary behaviour and risk of all-cause, cardiovascular and cancer mortality, and incident type 2 diabetes: a systematic review and dose response meta-analysis. Eur. J. Epidemiol..

[4] Caspersen CJ, Fulton JE (2008). Epidemiology of walking and type 2 diabetes. Med. Sci. Sports Exerc..

[5] Bouchard C, Blair SN, Katzmarzyk PT (2015). Less sitting, more physical activity, or higher fitness?. Mayo Clin. Proc..

[6] Mozaffarian D (2009). Lifestyle risk factors and new-onset diabetes mellitus in older adults: the cardiovascular health study. Arch. Intern. Med..

[7] Joseph JJ (2016). Physical activity, sedentary behaviors and the incidence of type 2 diabetes mellitus: the Multi-Ethnic Study of Atherosclerosis (MESA). BMJ Open Diabetes Res. Care.

[8] Colberg SR (2010). Exercise and Type 2 diabetes. Am. Coll. Sports Med. Am. Diabetes Assoc Joint Position Statement.

[9] Mendes R (2016). Exercise prescription for patients with type 2 diabetes-a synthesis of international recommendations: narrative review. Br. J. Sports Med..

[10] American Diabetes Association. 5. Prevention or Delay of Type 2 Diabetes: Standards of Medical Care in Diabetes-2018. *Diabetes care* **41**, S51–54. 10.2337/dc18-S005 (2018). 10.2337/dc18-S005 29222376

[11] Tsushita K (2018). Rationale and descriptive analysis of Specific Health Guidance: the nationwide lifestyle intervention program targeting metabolic syndrome in Japan. J. Atherosclerosis Thromb..

[12] Helmrich SP, Ragland DR, Leung RW, Paffenbarger RS (1991). Physical activity and reduced occurrence of non-insulin-dependent diabetes mellitus. New Engl. J. Med..

[13] Hu FB (1999). Walking compared with vigorous physical activity and risk of type 2 diabetes in women: a prospective study. JAMA.

[14] Nakao YM (2018). Effectiveness of nationwide screening and lifestyle intervention for abdominal obesity and cardiometabolic risks in Japan: the metabolic syndrome and comprehensive lifestyle intervention study on nationwide database in Japan (MetS ACTION-J study). PLoS ONE.

[15] Alberti KG (2009). Harmonizing the metabolic syndrome: a joint interim statement of the International Diabetes Federation Task Force on Epidemiology and Prevention; National Heart, Lung, and Blood Institute; American Heart Association; World Heart Federation; International Atherosclerosis Society; and International Association for the Study of Obesity. Circulation.

[16] Ainsworth BE (2000). Compendium of physical activities: an update of activity codes and MET intensities. Med. Sci. Sports Exerc..

[17] Jeon CY, Lokken RP, Hu FB, van Dam RM (2007). Physical activity of moderate intensity and risk of type 2 diabetes: a systematic review. Diabetes Care.

[18] Amagasa S (2018). Is objectively measured light-intensity physical activity associated with health outcomes after adjustment for moderate-to-vigorous physical activity in adults? A systematic review. Int. J. Behav. Nutr. Phys. Act..

[19] Syddall HE, Westbury LD, Cooper C, Sayer AA (2015). Self-reported walking speed: a useful marker of physical performance among community-dwelling older people?. J. Am. Med. Dir. Assoc..

[20] Ng SSM (2013). Assessing the walking speed of older adults: the influence of walkway length. Am. J. Phys. Med. Rehabil..

[21] Middleton A, Fritz SL, Lusardi M (2015). Walking speed: the functional vital sign. J. Aging Phys. Act..

[22] Kuwahara K (2014). Association of cardiorespiratory fitness and overweight with risk of type 2 diabetes in Japanese men. PLoS ONE.

[23] Codella R, Ialacqua M, Terruzzi I, Luzi L (2018). May the force be with you: why resistance training is essential for subjects with type 2 diabetes mellitus without complications. Endocrine.

[24] Centers for Disease Control and Prevention. Compliance with physical activity recommendations by walking for exercise--Michigan, 1996 and 1998. *MMWR. Morbidity and mortality weekly report* **49**, 560–565 (2000). 10921494

[25] Biddle SJ, Garcia Bengoechea E, Wiesner G (2017). Sedentary behaviour and adiposity in youth: a systematic review of reviews and analysis of causality. Int. J. Behav. Nutr. Phys. Act..

[26] Wirth K (2017). Biomarkers associated with sedentary behaviour in older adults: A systematic review. Ageing Res. Rev..

[27] Di Meo S, Iossa S, Venditti P (2017). Improvement of obesity-linked skeletal muscle insulin resistance by strength and endurance training. J. Endocrinol..

[28] Stamatakis E (2018). Self-rated walking pace and all-cause, cardiovascular disease and cancer mortality: individual participant pooled analysis of 50 225 walkers from 11 population British cohorts. Br. J. Sports Med..

[29] Martinez-Huenchullan S, McLennan SV, Verhoeven A, Twigg SM, Tam CS (2017). The emerging role of skeletal muscle extracellular matrix remodelling in obesity and exercise. Obes. Rev. Off. J. Int. Assoc. Study Obes..

[30] Tuomilehto J (2001). Prevention of type 2 diabetes mellitus by changes in lifestyle among subjects with impaired glucose tolerance. New Engl. J. Med..

[31] Lindström J (2006). Sustained reduction in the incidence of type 2 diabetes by lifestyle intervention: follow-up of the Finnish Diabetes Prevention Study. The Lancet.

[32] Group, D. P. P. R (2002). Reduction in the Incidence of type 2 diabetes with lifestyle intervention or metformin. N. Engl. J. Med..

[33] Kosaka K, Noda M, Kuzuya T (2005). Prevention of type 2 diabetes by lifestyle intervention: a Japanese trial in IGT males. Diabetes Res. Clin. Pract..

[34] Li G (2008). The long-term effect of lifestyle interventions to prevent diabetes in the China Da Qing Diabetes Prevention Study: a 20-year follow-up study. The Lancet.

[35] Ramachandran A (2006). The Indian diabetes prevention programme shows that lifestyle modification and metformin prevent type 2 diabetes in Asian Indian subjects with impaired glucose tolerance (IDPP-1). Diabetologia.

[36] Gillies CL (2007). Pharmacological and lifestyle interventions to prevent or delay type 2 diabetes in people with impaired glucose tolerance: systematic review and meta-analysis. BMJ (Clin. Res. ed.).

[37] Iseki K (2012). Risk factor profiles based on estimated glomerular filtration rate and dipstick proteinuria among participants of the specific health check and guidance system in Japan 2008. Clin. Exp. Nephrol..

[38] Wakasugi M (2014). Association between combined lifestyle factors and non-restorative sleep in Japan: a cross-sectional study based on a Japanese health database. PLoS ONE.

[39] Yano Y (2015). Long-term blood pressure variability, new-onset diabetes mellitus, and new-onset chronic kidney disease in the Japanese general population. Hypertension (Dallas, Tex).

[40] Kashiwagi A (2012). International clinical harmonization of glycated hemoglobin in Japan: From Japan Diabetes Society to National Glycohemoglobin Standardization Program values. J. Diabetes Investig..

[41] McLester CN, Nickerson BS, Kliszczewicz BM, McLester JR (2018). Reliability and Agreement of various InBody body composition analyzers as compared to dual-energy x-ray absorptiometry in healthy men and women. J. Clin. Densitom..

[42] Kim M, Shinkai S, Murayama H, Mori S (2015). Comparison of segmental multifrequency bioelectrical impedance analysis with dual-energy X-ray absorptiometry for the assessment of body composition in a community-dwelling older population. Geriatr. Gerontol. Int..

