# Supplementary material for: Fast walking is a preventive factor against new-onset diabetes mellitus in a large cohort from a Japanese general population
Source: Sci Rep. 2021 Jan 12;11:716. doi: 10.1038/s41598-020-80572-y (PMC7804125; doi:10.1038/s41598-020-80572-y)
Supplement: Supplementary file 2 — Supplementary Information. [file 41598_2020_80572_MOESM2_ESM.pdf]

Fast walking is a preventive factor against new-onset diabetes mellitus in a large cohort from a Japanese general population

Mariko Iwasaki, Akihiro Kudo, Koichi Asahi, Noritaka Machii, Kunitoshi Iseki, Hiroaki Satoh, Toshiki Moriyama, Kunihiro Yamagata, Kazuhiko Tsuruya, Shouichi Fujimoto, Ichiei Narita, Tsuneo Konta, Masahide Kondo, Yugo Shibagaki, Masato Kasahara, Tsuyoshi Watanabe, and Michio Shimabukuro

Additional File 2. Baseline characteristics of participants with or without fast walking in a subgroup

	Total	Fast walking –	Fast walking +	P
n	183	114	69	
Age, years	64.6 (12.6)	63.9 (13.2)	65.6 (11.4)	0.34
% Male	51.0	50.9	52.2	0.86
BMI, kg/m^2	26.7 (5.9)	27.6 (6.3)	25.4 (5.1)	0.02
Waist circumference, cm	91.9 (14.2)	94.1 (14.4)	88.5 (13.3)	0.01
Systolc blood pressure, mmHg	132.3 (16.6)	131.6 (16.6)	133.6 (17.5)	0.44
Diastolic blood pressure, mmHg	73.4 (10.6)	73.0 (10.5)	74.0 (10.7)	0.53
Fasting plasma glucose, mg/dl	138.9 (36.6)	136.2 (33.5)	143.3 (41.2)	0.20
HbA1c, %	7.17 (0.98)	7.14 (0.84)	7.22 (1.18)	0.59
LDL cholesterol, mg/dL	98.1 (25.2)	97.2 (24.1)	99.9 (27.0)	0.48
HDL cholesterol, mg/dL	57.9 (15.6)	56.9 (13.2)	59.5 (18.8)	0.28
Triglycerides, mg/dL	125.0 (154.6)	140.8 (190.6)	99.0 (50.3)	0.03
AST,U/L	22.4 (9.1)	24.9 (12.9)	22.4 (9.1)	0.78
ALT, U/L	20.6 (13.7)	23.3 (13.6)	20.6 (13.7)	0.94
Every day drinking, %	25.4	18.4	20.3	0.53
Current smoker, %	19.7	19.3	20.3	0.86
Weight gain over 10kg since 20 years of age, %	61.7	62.3	60.9	0.85
Exercise to sweat lightly, %	26.2	16.7	42.0	0.00
Walking>1 hour/day, %	38.8	28.9	55.1	0.00
Weight change ± 3kg within 1year, %	47.5	50.9	42.0	0.24
10m walking, sec	6.75 (1.84)	7.08 (2.11)	6.20 (1.11)	0.00

Mean (SD) or %
