# Supplementary material for: Fast walking is a preventive factor against new-onset diabetes mellitus in a large cohort from a Japanese general population
Source: Sci Rep. 2021 Jan 12;11:716. doi: 10.1038/s41598-020-80572-y (PMC7804125; doi:10.1038/s41598-020-80572-y)
Supplement: Supplementary file 3 — Supplementary Information. [file 41598_2020_80572_MOESM3_ESM.pdf]

Fast walking is a preventive factor against new-onset diabetes mellitus in a large cohort from a Japanese general population

Mariko Iwasaki, Akihiro Kudo, Koichi Asahi, Noritaka Machii, Kunitoshi Iseki, Hiroaki Satoh, Toshiki Moriyama, Kunihiro Yamagata, Kazuhiko Tsuruya, Shouichi Fujimoto, Ichiei Narita, Tsuneo Konta, Masahide Kondo, Yugo Shibagaki, Masato Kasahara, Tsuyoshi Watanabe, and Michio Shimabukuro

Additional File 3. Baseline characteristics of participants with or without exercise to sweat lightly

	Exercise to sweat lightly –	Exercise to sweat lightly +	*P*
n	97,770	69,914	
Age, years	62.4(8.3)	65.5(6.6)	<0.01
Male, %	35.9	42.8	<0.01
BMI, kg/m^2	23.0(3.2)	23.0(2.9)	N.S.
Waist circumference, cm	83.3(9.1)	83.2(8.4)	N.S.
Systolc blood pressure, mmHg	128.4(17.6)	129.8(17.2)	<0.01
Diastolic blood pressure, mmHg	76.3(10.8)	76.5(10.4)	<0.01
Fasting plasma glucose, mg/dl	93.1(9.7)	93.7(9.8)	<0.01
HbA1c, %	5.58(0.33)	5.59(0.33)	<0.01
LDL cholesterol, mg/dL	126.8(30.1)	126.2(29.2)	<0.01
HDL cholesterol, mg/dL	62.4(15.9)	63.1(16.1)	<0.01
Triglycerides, mg/dL	114.2(72.3)	110.4(65.5)	<0.01
AST,U/L	23.9(9.4)	24.1(8.8)	<0.01
ALT, U/L	21.5(13.0)	20.9(11.5)	<0.01
ɤGTP, U/L	34.7(41.8)	33.6(38.7)	<0.01
Current smoker, %	14.8	10.6	<0.01
Every day drinking, %	20.8	23.8	<0.01
Weight gain over 10kg from twenty,%	31.7	30.5	<0.01
Weight change ± 3kg within 1year, %	20.5	19.0	<0.01

Mean (SD) or %, N.S. not significant
