# Supplementary material for: Fast walking is a preventive factor against new-onset diabetes mellitus in a large cohort from a Japanese general population
Source: Sci Rep. 2021 Jan 12;11:716. doi: 10.1038/s41598-020-80572-y (PMC7804125; doi:10.1038/s41598-020-80572-y)
Supplement: Supplementary file 5 — Supplementary Information. [file 41598_2020_80572_MOESM5_ESM.pdf]

Fast walking is a preventive factor against new-onset diabetes mellitus in a large cohort from a Japanese general population

Mariko Iwasaki, Akihiro Kudo, Koichi Asahi, Noritaka Machii, Kunitoshi Iseki, Hiroaki Satoh, Toshiki Moriyama, Kunihiro Yamagata, Kazuhiko Tsuruya, Shouichi Fujimoto, Ichiei Narita, Tsuneo Konta, Masahide Kondo, Yugo Shibagaki, Masato Kasahara, Tsuyoshi Watanabe, and Michio Shimabukuro

Additional File 5. Characteristics at baseline or at follow-up or onset of diabetes

	Diabetes onset –			Diabetes onset +		
	Baseline	At follow-up	*P*	Baseline	At onset	*P*
n	161,455			6,229		
Age, years	63.6 (7.8)	65.6 (7.7)	<0.05	65.2 (6.5)	66.8 (6.4)	<0.05
BMI, kg/m^2	22.9 (3.1)	22.9 (3.1)	0.053	24.3 (3.5)	24.6 (3.6)	<0.05
Waist circumference, cm	83.1 (8.8)	83.1 (8.8)	0.47	86.8 (9.1)	87.4 (9.4)	<0.05
Fasting plasma glucose, mg/dl	92.9 (9.3)	93.0 (9.8)	<0.05	105.1 (11.6)	118.0 (23.4)	<0.05
HbA1c, %	5.57 (0.32)	5.59 (0.32)	<0.05	6.01 (0.34)	6.35 (0.64)	<0.05

Mean (SD) or %
