# Supplementary material for: Fast walking is a preventive factor against new-onset diabetes mellitus in a large cohort from a Japanese general population
Source: Sci Rep. 2021 Jan 12;11:716. doi: 10.1038/s41598-020-80572-y (PMC7804125; doi:10.1038/s41598-020-80572-y)
Supplement: Supplementary file 6 — Supplementary Information. [file 41598_2020_80572_MOESM6_ESM.pdf]

Fast walking is a preventive factor against new-onset diabetes mellitus in a large cohort from a Japanese general population

Mariko Iwasaki, Akihiro Kudo, Koichi Asahi, Noritaka Machii, Kunitoshi Iseki, Hiroaki Satoh, Toshiki Moriyama, Kunihiro Yamagata, Kazuhiko Tsuruya, Shouichi Fujimoto, Ichiei Narita, Tsuneo Konta, Masahide Kondo, Yugo Shibagaki, Masato Kasahara, Tsuyoshi Watanabe, and Michio Shimabukuro

Additional File 6. Multivariable-adjusted odds ratio for the risk of new-onset diabetes mellitus of fast walking

	Weight gain over 10kg from twenty				Weight change ± 3kg within 1year			
	–		+		–		+	
	OR* (95% CI)	*P*	OR* (95% CI)	*P*	OR* (95% CI)	*P*	OR* (95% CI)	*P*
Fast walking	0.91 (0.85-0.99)	<0.05	0.90 (0.83-0.98)	<0.05	0.90 (0.85-0.96)	<0.05	0.92 (0.83-1.03)	0.16

*Adjusted for sex, age, BMI, SBP, FPG, exercise to sweat lightly, walking>1 hour/day
